# Photodegradation of a bacterial pigment and resulting hydrogen peroxide release enable coral settlement

**DOI:** 10.1038/s41598-023-30470-w

**Published:** 2023-03-02

**Authors:** Lars-Erik Petersen, Matthias Y. Kellermann, Laura J. Fiegel, Samuel Nietzer, Ulf Bickmeyer, Doris Abele, Peter J. Schupp

**Affiliations:** 1 Institute for Chemistry and Biology of the Marine Environment (ICBM), Carl-von-Ossietzky University Oldenburg, Schleusenstrasse 1, 26382 Wilhelmshaven, Germany; 2 Alfred Wegener Institute Helmholtz Center for Polar and Marine Research (AWI), Am Handelshafen 12, 27570 Bremerhaven, Germany; 3 Helmholtz Institute for Functional Marine Biodiversity (HIFMB) at the University of Oldenburg, Ammerländer Heerstrasse 231, 26129 Oldenburg, Germany; 4 Present Address: Department of Plant and Environmental Sciences, Weizmann Institute of Science, 7610001 Rehovot, Israel

**Keywords:** Restoration ecology, Tropical ecology, Ecology, Environmental sciences, Environmental chemistry

## Abstract

The global degradation of coral reefs is steadily increasing with ongoing climate change. Yet coral larvae settlement, a key mechanism of coral population rejuvenation and recovery, is largely understudied. Here, we show how the lipophilic, settlement-inducing bacterial pigment cycloprodigiosin (CYPRO) is actively harvested and subsequently enriched along the ectoderm of larvae of the scleractinian coral *Leptastrea purpura*. A light-dependent reaction transforms the CYPRO molecules through photolytic decomposition and provides a constant supply of hydrogen peroxide (H_2_O_2_), leading to attachment on the substrate and metamorphosis into a coral recruit. Micromolar concentrations of H_2_O_2_ in seawater also resulted in rapid metamorphosis, but without prior larval attachment. We propose that the morphogen CYPRO is responsible for initiating attachment while simultaneously acting as a molecular generator for the comprehensive metamorphosis of pelagic larvae. Ultimately, our approach opens a novel mechanistic dimension to the study of chemical signaling in coral settlement and provides unprecedented insights into the role of infochemicals in cross-kingdom interactions.

## Introduction

Profound changes in coral reef communities are recognized at increasing frequency and severity, often resulting in strong declines in biodiversity and ecosystem functioning. As climate change continues, the destruction of coral reefs is expected to worsen, with serious consequences for the livelihoods of several hundred million people^1–7^. Adult corals in particular have shown to be more vulnerable to climate change, while juveniles appear to possess a broader physiological plasticity, in part due to their greater flexibility in exchanging beneficial symbiotic dinoflagellates^8–10^. Thus, the solution for fighting coral decline may not be found in the parent population but rather in the recruitment of juvenile coral generations, capable to adapt to constantly fluctuating conditions in a rapidly changing Anthropocene. Sexual reproduction enables scleractinian corals to generate myriads of pelagic larvae that must recruit onto suitable substrates to achieve their permanent sessile life stage. This process – i.e*.*, coral settlement – is characterized by the attachment to a desired surface followed by a rapid metamorphic event that transforms larvae into sessile, benthic recruits. Current research has intensified its focus on the early life-stages of corals and highlights the idea of inductive cues as a requirement to induce the cascade of attachment and subsequent metamorphosis in coral larvae. These cues can be of varying nature and include, for example, light^11–13^, reef sound^14^ and surface structure^15^. However, many studies have proven the efficacy of live crustose coralline algae (CCA) as biological, settlement-inducing substrate for larvae of various coral species^16–18^. Moreover, microbial mats, covering the surface of marine hard substrates such as CCA, but also monospecific bacterial biofilms have received considerable attention as potent coral settlement inducers^19–24^. Particularly the genus Pseudoalteromonas features a variety of species capable of stimulating settlement in coral larvae and other marine invertebrates^19,23,25–29^. These insights greatly improved todays understanding of coral settlement, but we are still missing mechanistic insights into this morphogenic reaction on the molecular level. In rare cases, the inductive activity towards coral recruits was attributed to a mixture of chemical molecules produced by the CCA holobiont^30–32^. Isolated coral settlement inducing compounds from CCA or associated microorganisms often remain poorly described^26,33^ and only a few have been fully characterized^34–36^. To date, there are only two settlement inducing compounds derived from bacteria described in the literature: tetrabromopyrrole (TBP) and cycloprodigiosin (CYPRO). TBP is a brominated secondary metabolite isolated from the CCA-associated *Pseudoalteromonas sp.* PS5 strain, that induced metamorphosis (mostly without prior attachment) of Pacific *Acropora millepora* larvae^25^, but complete settlement of Caribbean *Porites astreoides*, *Orbicella franksi*, *A. palmata* and Pacific *Leptastrea purpurea* larvae at varying levels^28,37^. CYPRO is a reddish alkaloidal pigment that was isolated along with other prodiginines from the CCA-associated bacterium *Pseudoalteromonas rubra* #1783. It induced complete settlement of *L. purpurea* larvae in a light- and concentration dependent manner^29^. Although TBP and CYPRO are chemically fully defined, both compounds lack further functional characterization in the coral settlement process and their mode of action remains unknown. Thus, the present study sought to investigate the interaction between the settlement cue CYPRO and coral larvae and, in particular, to decipher possible molecular traits that explain the efficacy of CYPRO. Building on our previous work^23,29,37–40^, we here provide an unprecedented visualization of the uptake of a chemical settlement inducer by coral larvae and reveal an underlying molecular mechanism that promotes the complex transformation of pelagic larvae to sessile recruits. Ultimately, our study provides unique insights into the role of infochemicals in coral reproductive biology and opens a chemo-mechanistic element in the study of coral settlement.

## Results

In our previous studies, we isolated the settlement inducing, Gram-negative, red-pigmented bacterial strain *Pseudoalteromonas rubra* #1783 from the CCA *Hydrolithon reinboldii*^23^. Subsequently, bioassay-guided fractionation of the crude extract of *P. rubra* #1783 led to the identification of CYPRO as the responsible bioactive molecule that triggered settlement in a light-dependent manner in less than 48 h at high success rates (approaching 90% settlement)^29^. Here, we investigate the uptake mechanism of CYPRO in *L. purpurea* larvae and further reveal a possible mode of action for the photosensitive morphogenic cue.

### Active acquisition of CYPRO by *L. purpurea* larvae

**Figure 1:**
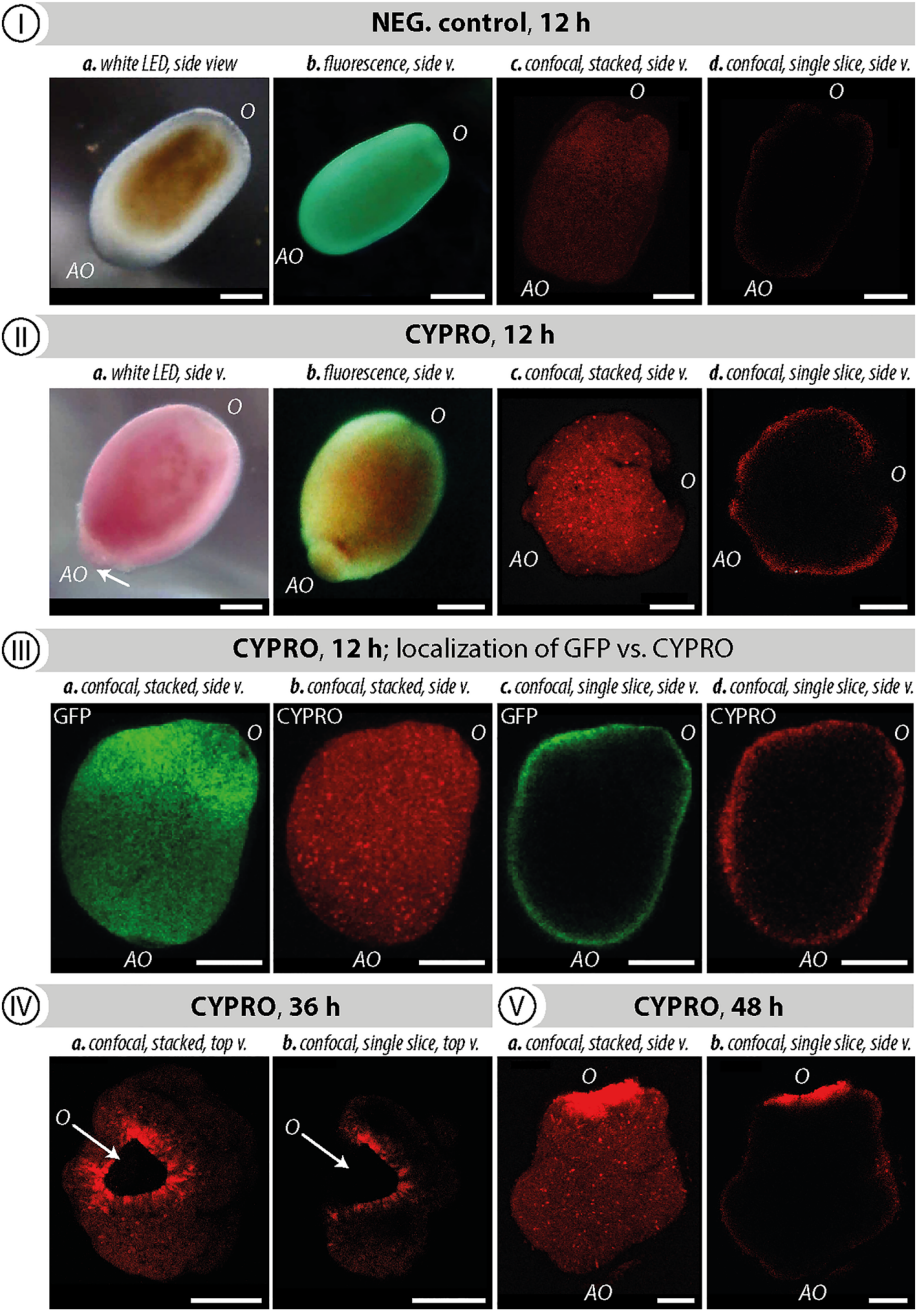
Cycloprodigiosin (CYPRO) uptake by *Leptastrea purpurea* larvae examined for 48 h in the absence of light. (**I**) Swimming larva without CYPRO (negative control), (**II**) metamorphosing larva 12 h after CYPRO addition (0.2 µg mL^−1^) and (**III**) a location-based comparison between CYPRO in red and green fluorescence protein (GFP) in green, (**IV**) 36 h and (**V**) 48 h after CYPRO addition. Fluorescence emitted by larvae and their symbionts was captured using light microscopy combined with blue light and yellow filters (**I**-a,b, **II**-a,b) and confocal laser scanning fluorescence microscopy (**I**-c,d, **II**-c,d, **III**-V). Confocal pictures are displayed as stacked (n = 50) and single shots (n = 1 taken from the center of larva). An excitation wavelength of 514 nm and an emission window of 550–570 nm was used for CYPRO detection, while GFPs were visualized by an excitation wavelength of 488 nm and an emission window of 500–520 nm. Scale bars: 250 μm.

Figure 1 illustrates the active uptake and allocation of CYPRO by *L. purpurea* larvae within 48 h. Since CYPRO is light sensitive, this experiment has been conducted in complete darkness to prevent premature photobleaching of the pigment. After 12 h of exposure to CYPRO (1 μg in 5 mL filtered artificial seawater (FASW); 0.2 µM PTFE filters, VWR International, Radnor, PA, United States) the appearance of swimming or recently attached coral larvae changed from translucent (Fig. 1 I -a,b) to a prominent dark-red color (Fig. 1 II-a,b). Negative control larvae, *i.e.* no added CYPRO, remained translucent and motile throughout the experiment and retained this property for longer than 1 month. Confocal laser scanning fluorescence microscopy further surveyed the internal distribution of the fluorescent cue CYPRO in *L. purpurea* larvae during settlement after 12, 36 and 48 h (Fig. 1 I–V). Single shots were taken from the center of a stack (z-axis), whereas stacked pictures represent an overlay of approximately 50 single shots, creating a projection image of the larva. Negative control larvae showed only weak signal intensity, representing background noise (Fig. 1 I-c,d). The highly hydrophobic molecule CYPRO was at first collected from the well bottom and subsequently enriched within the outer surface of coral larvae (Fig. 1 II-c,d). During metamorphosis at 36 and 48 h post attachment, the morphogenic pigment was predominantly detected around the oral section of transforming larvae (Fig. 1 IV-a,b and V-a,b). At the same time, a widening of the oral disk as well as first signs of mesentery development were noticeable. Comparing the emission patterns of CYPRO with those of green fluorescent proteins (GFPs), a protein regularly biosynthesized in many coral species^37,40–42^, showed that both compounds are located within the same tissue region after 12 h of cue exposure (Fig. 1 III -a,b,c,d). Noteworthy, excitation of CYPRO at 514 nm did not excite GFP and thus allowed clear discrimination of emission spectra for both compounds (*cf.* Figure S1).

### Photolytic decomposition of CYPRO and subsequent H_2_O_2_ production

**Figure 2:**
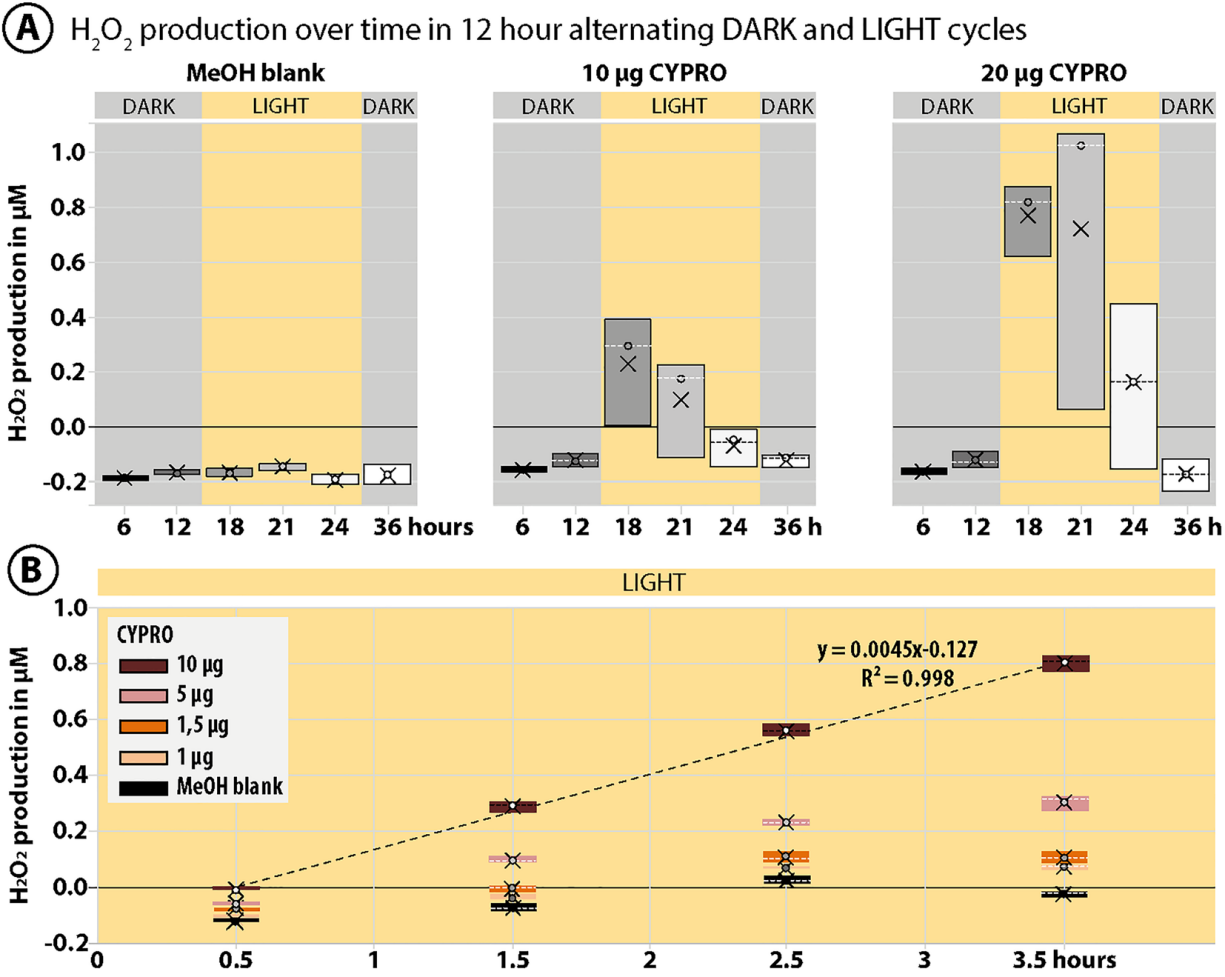
Hydrogen peroxide (H_2_O_2_) production by cycloprodigiosin (CYPRO) over time in oscillating light regimes. (**A**) Production of H_2_O_2_ by photodegradation of CYPRO at 10 and 20 μg in 300 μL filtered artificial seawater (FASW). H_2_O_2_ concentrations were monitored for 36 h in 12 h-alternating phases of darkness (grey) and light (yellow). (**B**) Light-dependent linear production of H_2_O_2_ by 1, 1.5, 5 and 10 μg CYPRO in 300 μL FASW within the first 3.5 h of exposure to light after darkness. The light driven degradation of CYPRO clearly corresponds with a constant and linear production of H_2_O_2_. Evaporated MeOH without CYPRO served as blanks. In the boxplot, the dashed lines indicate the median, while X represents the mean H2O2 production at each time point.

As reported in our previous study^29^, the pigment CYPRO is extremely unstable in the presence of oxygen and light. Here, we further reveal that the light driven degradation of CYPRO^29^ (*cf.* Figure S2) clearly corresponds with a constant and linear production of the reactive oxygen species (ROS) hydrogen peroxide (H_2_O_2_) (Fig. 2A,B). Originally, applying a 12 h darkness-light cycle in combination with CYPRO yielded high settlement success rates (approaching 90 %) in *L. purpurea* larvae^29^. Thus, the production of H_2_O_2_ by different amounts of CYPRO (10 and 20 μg) was monitored in similar oscillating light regimes (Figure 2A). Concentrations of H_2_O_2_ produced by 10 and 20 μg CYPRO did not increase within the first 12 h of darkness. However, after 6 h of light exposure (18 h total experiment time) H_2_O_2_ concentrations rose up to 0.3 and 0.8 μM for 10 and 20 μg CYPRO, respectively. Continuing light stress for another 3 and 6 h (i.e*.*, 21 and 24 h total experiment time) did not enhance ROS production but in fact lowered H_2_O_2_ concentrations back to 0 μM due to complete photodegradation of CYPRO over time (*cf.* Figure S2) and the general unstable character of H_2_O_2_ in that system. The negative control (blank consisting of MeOH without CYPRO) did not produce significant amounts of H_2_O_2_ over the entire duration of the experiment. A second experiment was performed to follow H_2_O_2_ production by CYPRO during the first crucial hours of constant light exposure (Fig. 2B). Testing four different amounts of CYPRO (1, 1.5, 5 and 10 μg) in close intervals for 3.5 h resulted in a linear increase of H_2_O_2_ concentrations over time. This linearity in production was most pronounced for 10 μg CYPRO that generated 0.8 μM H_2_O_2_ after 3.5 h of light exposure. Lower CYPRO amounts (i.e*.*, 1, 1.5 and 5 μg) showed a linear increase in H_2_O_2_ concentrations only for the first three time points – likely, the pigment had fully decomposed at the final sampling point (Fig. 2B, Figure S2).

### H_2_O_2_-induced `nonsense metamorphosis’

**Figure 3:**
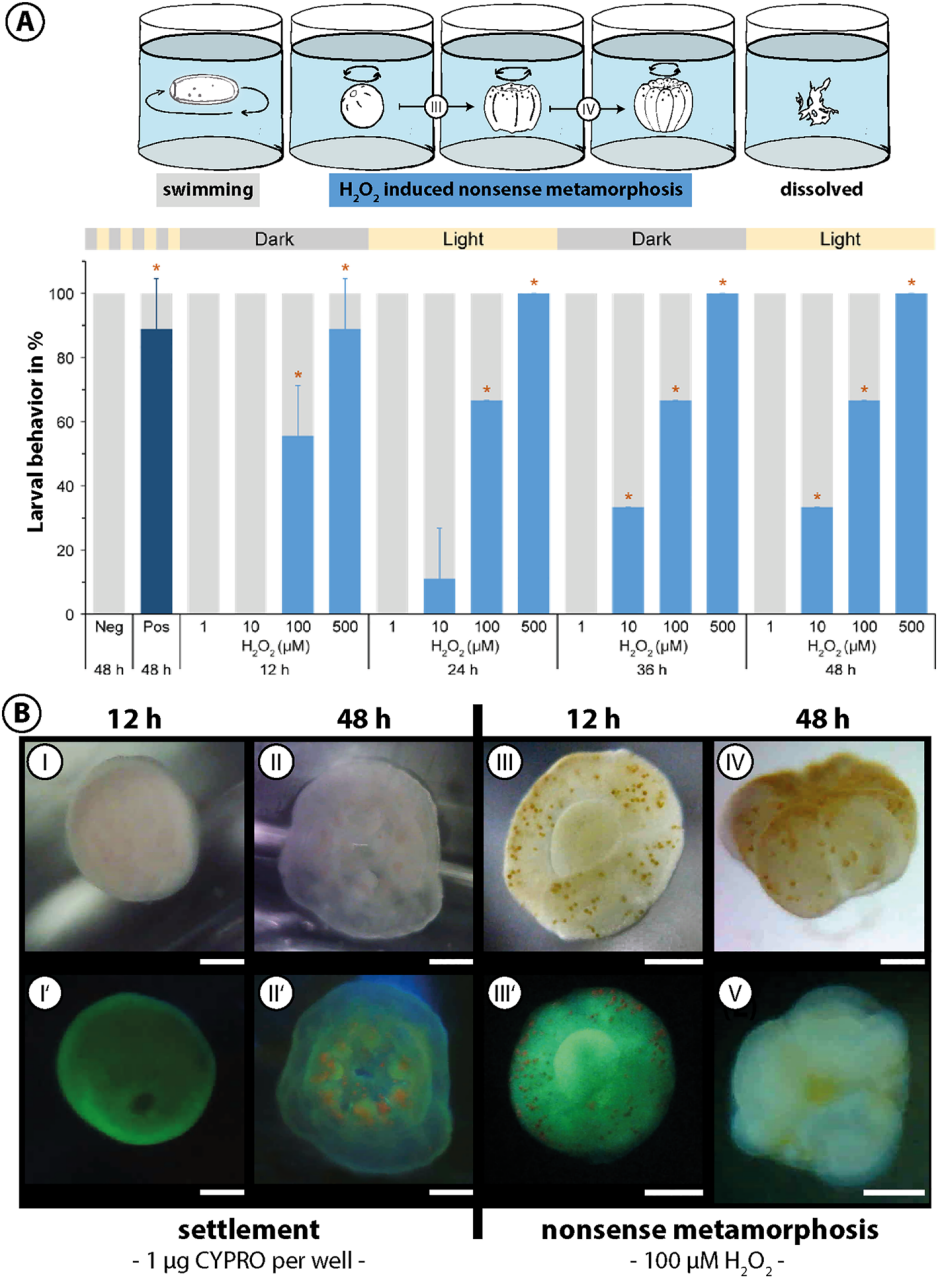
Hydrogen peroxide (H_2_O_2_) induces `nonsense metamorphosis’ in *Leptastrea purpurea* larvae. (**A**) Settlement behavior of larvae (n = 3 wells) exposed to 1, 10, 100 and 500 µM H_2_O_2_ during 12 h-alternating phases of darkness and light. Percentages of swimming larvae are displayed in light grey, ‘nonsense metamorphosis’ in light blue and settlement (i.e*.,* attachment and metamorphosis) in dark blue. 0.2 µg mL^−1^ cycloprodigiosin (CYPRO) served as settlement cue (positive control, Pos) and equivalent amounts of evaporated MeOH as negative control (Neg). Error bars show standard deviations and asterisks represent significant responses compared to negative control (*p* < 0.05). (**B**) Different morphologies of *L*. *purpurea* larvae exposed to either 0.2 µg mL^−1^ CYPRO (I, II) or 100 µM H_2_O_2_ (III, IV) for 12 and 48 h. Fluorescence emitted by larvae and their symbionts was captured using light microscopy combined with blue light and yellow filters (I’, II’, III’) and confocal laser scanning fluorescence microscopy (IV’). Scale bars: 250 μm.

The linear relationship between CYPRO photodegradation and H_2_O_2_ production (*cf.* Fig. 2B) stimulated the idea of a follow-up experiment, investigating the direct effect of μM-levels H_2_O_2_ on the settlement behavior of *L. purpurea* larvae and testing light as secondary cue in this setup. Four concentrations of H_2_O_2_ (1, 10, 100, 500 μM) were used to test its effect on coral larvae over 48 h under oscillating light regimes (Figure 3A). In contrast to the positive control CYPRO (0.2 μg mL^−1^), inducing settlement at 88.9% ± 15.7 (*p* = 0.025) within 48 h, larvae exposed to H_2_O_2_ only performed morphological changes similar to natural metamorphosis, but without prior attachment. This phenomenon was eventually fatal to floating primary polyps – and therefore termed `nonsense metamorphosis’. After 12 h in darkness, 88.9% ± 15.7 (*p* = 0.034) of coral larvae elicited `nonsense metamorphosis’ in response to 500 μM H_2_O_2_ while it was 55.6% ± 15.7 (*p* = 0.034) in response to 100 μM. Since light was completely absent during the initial 12 h of this experiment and larvae still underwent `nonsense metamorphosis’ in response to 500 and 100 μM H_2_O_2_, a possible role of light as a secondary cue can be excluded in this process. `Nonsense metamorphosis’ rates increased to 100% ± 0 (*p* = 0.025) and 66.7% ± 0 (*p* = 0.025) for 500 and 100 μM H_2_O_2_ after 24 h, respectively, and remained at this level until the end of the experiment (48 h). The first signs of `nonsense metamorphosis’ for 10 µM H_2_O_2_ appeared after 24 h (11.1% ± 15.7) and rose to significant levels (33.3% ± 0, *p* = 0.025) after 36 h. 1 µM H_2_O_2_ did not cause ‘nonsense metamorphosis’ in *L. purpurea* larvae throughout the experimental duration. Larvae that did not show any settlement behavior to the here tested H_2_O_2_ concentrations remained unaffected and continued swimming. The different settlement behaviors induced by either CYPRO or H_2_O_2_ (settlement vs. ‘nonsense metamorphosis’, respectively) were accompanied by distinct larval morphologies for both morphogens (Fig. 3B). Coral larvae exposed to 0.2 μg mL^−1^ CYPRO in oscillating light regimes attached within 12 h (Figure 3B-I, I’) and developed their typical “flower-like” shape with pronounced oral disks, six constrictions as well as clear signs of mesentery and tentacle development after 48 h (Fig. 3B-II, II’). In contrast, 100 µM H_2_O_2_ elicited rapid perceivable transformations in larval morphology within 12 h (Fig. 3B-III, III’). Coral larvae proceeded their ‘nonsense metamorphosis’ and 48 h after exposure to H_2_O_2_ they had developed an irregular “flower-like” shape while drifting in the water, rotating around their own axis (Fig. 3B-IV, IV’). Ultimately, ‘nonsense metamorphosis’ led to death and disintegration of *L. purpurea* larvae after approximately 1 week of experimental time while larvae exposed to CYPRO continued to develop into functional coral polyps (Figure S3).

## Discussion

**Figure 4:**
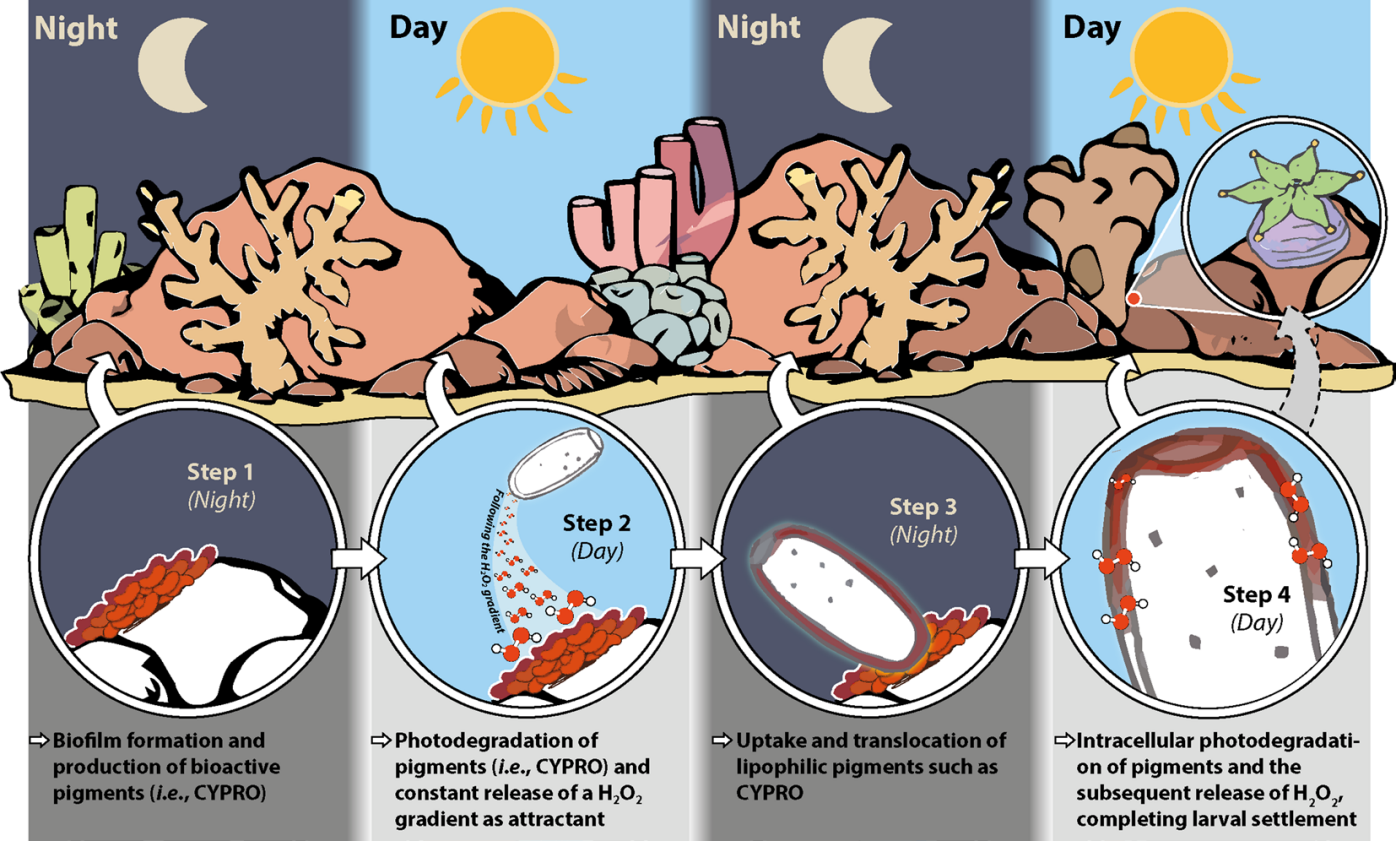
Conceptual model of cycloprodigiosin (CYPRO) and H_2_O_2_ induced settlement in coral larvae. Microbial biofilms consisting of settlement-inducing bacteria such as *Pseudoalteromonas rubra* assemble on marine hard substrates and continue to produce bioactive pigments like CYPRO (**Step 1**). During phases of high light intensity, the photosensitive pigments bleach and establish an H_2_O_2_ gradient that attracts coral larvae (**Step 2**). Following the gradient, coral larvae encounter, accumulate and utilize intact lipophilic pigments for the following transformation (**Step 3**). Eventually, the light-driven degradation of accumulated pigments releases elevated intracellular levels of H_2_O_2_ that seems to be crucial for the transformation into coral recruits (**Step 4**) and eventually juvenile polyps.

We have demonstrated that the lipophilic pigment CYPRO produced by the CCA-associated bacterium *Pseudoalteromonas rubra* #1783 triggers settlement in larvae of the stony coral *Leptastrea purpurea*. It does so by being actively collected and subsequently enriched within the outer larval surface tissue. We further revealed that the light-driven degradation of CYPRO in attached larvae is accompanied by a steady production of µM levels H_2_O_2_, resulting in metamorphosis of the larvae on the substrate. µM levels of H_2_O_2_ added to seawater induced only rapid ‘nonsense metamorphosis’ – a phenomenon bearing mild resemblance to natural metamorphosis, but without prior attachment and being eventually fatal to coral larvae. Furthermore, H_2_O_2_ rapidly induced ‘nonsense metamorphosis’ within 12 h of complete darkness, excluding light as a secondary cue in this morphogenic reaction. The here presented results suggest that the physico-chemical properties of CYPRO allow its active uptake and translocation along the outer tissue of coral larvae. Other prodiginines, a family of highly bioactive pigmented alkaloids of which CYPRO is a member^43,44^, such as prodigiosin and 2-methyl-3-hexyl prodiginine did not induce settlement in *L. purpurea* and in turn were toxic in a concentration-dependent manner^29^. In comparison, CYPRO lacks the hydrophobic alkyl chain and instead contains a six-membered ring, fused to the alkyl pyrrole (Figure S2). We propose that this structural feature could affect the uptake and solubility of CYPRO in the larval bilayer membrane – similarly to highly membranophilic quinones, carotenoids and squalenes^45^. Yet, the dominant presence of CYPRO at the oral section of a settling larva could also be a side effect of active tissue rearrangement during larval metamorphosis^46^. Nevertheless, our findings are intriguing since CYPRO was detected in the same location as GFP, which recently has been identified to fine-tune the light microclimate in corals^47^ and reported to scavenge reactive oxygen species (ROS) such as H_2_O_2_^48^. Although it is not trivial to investigate, possible interactions between both compounds cannot be excluded. In fact, interactions between photosensitive ROS-producing pigments like CYPRO and ROS-scavenging GFPs are likely to occur under certain light conditions and could correlate with the settlement success of coral larvae. We here suggest that the distinct cascade of events, *i.e.,* active uptake, translocation, molecular utilization and successive H_2_O_2_ production through photodegradation is a key factor for the effectiveness of CYPRO as a settlement inducer. In comparison to water-soluble H_2_O_2_, CYPRO is highly hydrophobic and thus actively harvested by the larvae from the well bottom (Fig. 1). Upon light exposure, the absorbed pigment is locally converted to H_2_O_2_, and larval attachment and metamorphosis are carried out consecutively^29^. In contrast, directly applied H_2_O_2_ can be homogeneously encountered throughout the entire well volume. Thus, µM additions of H_2_O_2_ (Fig. 3) induced metamorphosis in coral larvae, but only in the water column and without prior attachment (‘nonsense metamorphosis’), likely, since larvae do not encounter high local H_2_O_2_ concentrations as in the case of CYPRO. That is, our results contrast recent studies that reported synergistic effects of multiple morphogens, often of lipid and polysaccharide origin, in corals and other marine model organisms^26,49–52^. However, their mechanisms have not been described and it is debatable if the latter compounds are general metamorphic cues for cnidarian larvae. Conversely, there is supporting evidence for CYPRO-induced settlement via ROS production, since H_2_O_2_ has been shown to elicit settlement in various larvae of Caribbean scleractinian corals^53^ and here in Pacific corals as well. Since H_2_O_2_ is involved in the regulation of many reproductive processes^54–56^ it is tempting to examine whether the cascading photochemical production of ROS by chromophoric compounds such as CYPRO is a phenomenon with widespread ecological significance in coral settlement. These considerations led us to propose a conceptual model that incorporates insights from the current and past studies^23,29,39,40^ (Fig. 4). Microbial biofilms composed of settlement-inducing bacteria such as Pseudoalteromonas assemble on marine hard substrates and continue to produce photoactive chemicals such as the pigment CYPRO. During phases of high light intensity, the photosensitive molecules degrade and may establish an H_2_O_2_ gradient that attracts coral larvae. Following the gradient, coral larvae encounter, accumulate and utilize intact lipophilic compounds such as CYPRO for the subsequent transformation. Eventually, the light-driven degradation of accumulated pigments releases elevated intracellular levels of H_2_O_2_ that likely energize the costly metamorphosis into coral recruits. In the reef environment, additional morphogens and environmental parameters such as light quality and intensity might further diversify recruitment mechanisms and enhance settlement success rates of coral larvae. In summary, our findings let us hypothesize that the metamorphic cue CYPRO may act as a molecular battery which, through successive photodegradation, can facilitate a constant intracellular supply of elevated H_2_O_2_ levels over an extended period within the larval tissue, ultimately fueling the metamorphosis of *L. purpurea* larvae. If this chemical interaction indeed happens in vivo remains yet to be tested, however, our data likely supports such a scenario and provides a conceptual framework for future investigations. Accordingly, we substantiate the potential of bioactive settlement cues and encourage future coral conservation efforts to be mindful of external physical factors. This study has revealed the intricate interplay between organisms, biomolecules and abiotic factors, and has demonstrated that the photodegradation of a reactive compound is indeed the driving force behind the rate-limiting conversion of coral larvae to polyps. More research is needed to assess the ecological significance of CYPRO across scleractinian diversity, but preliminary data with *Acropora tenuis* larvae indicated settlement with a broadcast spawning species as well. Our approach is opening an unprecedented mechanistic element to the study of chemical signaling in coral larvae settlement and potentially other marine invertebrates.

## Methods

### Bacterial cultivation and compound isolation

*Pseudoalteromonas rubra* #1783 was originally isolated from the CCA *Hydrolithon reinboldii* that was collected between April and July 2010 in Luminao Reef, Guam, USA (13°27′53.57″ N, 144°38′54″ E). Isolation and phylogenetic identification of strain #1783 were conducted as described in Petersen and colleagues^29^. The settlement assay-guided purification and identification of CYPRO from *P. rubra* #1783 was performed as described in Petersen et al^29^. Briefly, *P. rubra* #1783 was grown on marine broth agar plates for 24 h at 27 °C, followed by scraping of the resulting bacterial lawn and extraction of the cell paste with ethanol (EtOH). The resulting, settlement inducing crude extract was initially separated by liquid-liquid partitioning using hexane, dichloromethane (DCM), ethyl acetate, and water (H_2_O). The active DCM fraction was further subjected to solid-phase extraction (reverse-phase, using a C_18_ SPE column) and sequentially eluted with a gradient of H_2_O and acetonitrile (ACN). The active fraction eluted with 100% ACN and was further purified on a preparative HPLC-DAD system (Agilent Technologies, Inc., Santa Clara, United States) using a semi-preparative reverse-phase C_18_ column to obtain pure CYPRO. High resolution single mass and MS/MS spectral data of CYPRO were measured on an ACQUITY Ultra Performance Liquid Chromatography (UPLC) H-Class System (Waters Co., Milford, MA, United States) coupled to a Synapt G2-Si HDMS high-resolution Q-ToF-MS (Waters Co., Manchester, United Kingdom) equipped with a LockSpray dual electrospray ion source operated in positive (POS) ionization mode. The mass spectrometer system was controlled by MassLynx™ software (version 4.2, Waters Corporation, Milford, MA, United States). Chromatographic separation was achieved on a Waters Acquity BEH C_18_ column (1.7 μm, 2.1 mm × 50 mm).

### Acquisition of coral larvae

Adult colonies of the brooding coral *L. purpurea* were used to obtain larvae. Initial brood stock of *L. purpurea* were collected from Luminao reef, Guam, in 2013. Released larvae settled on CCA (*H. reinboldii*) chips at the University of Guam Marine Laboratory. The resulting recruits were reared for 4 months and subsequently transported to the aquarium facilities of ICBM in Wilhelmshaven, Germany (CITES 13US06569B/9). The research aquarium is run with artificial seawater (Tropic Marin Pro Reef salt, Prof. Dr. Biener GmbH, Wartenberg, Germany). Reaching maturity at the age of approx. 2 years, these colonies started to release planulae and allowed a steady and daily collection of larvae in our aquarium facilities^37^. These planulae contain a green fluorescent protein (GFP) that allows easy detection using blue light (460–480 nm wavelengths) and yellow barrier filters (BlueStar, NIGHTSEA, Lexington, MA, United States)^40^. Since *L. purpurea* larvae are up to 1 mm in length, they were picked with a plastic pipette without further magnification. Larvae were collected in small glass flasks and could be kept in FASW for several weeks if water exchange was conducted weekly.

### Hydrogen peroxide (H_2_O_2_) settlement assays

In a previous study, we demonstrated that the combination of CYPRO at a concentration of 0.2 µg mL^−1^ and a dark-light rhythm (12 h per light phase, starting with darkness) lead to settlement rates in *L. purpurea* larvae approaching 90%^29^. Here, settlement assays were conducted in flat-bottom 12-well plates (TPP Techno Plastic Products AG, Trasadingen, Switzerland) and CYPRO was used as positive control. For this purpose, a methanol (MeOH) stock solution of 0.1 mg/mL solution of the morphogen was applied to the well bottoms using a target concentration of 0.2 µg mL^−1^ per each added mL of FASW (filtered artificial seawater, 0.2 µM PTFE filters, VWR International, Radnor, PA, United States). A comparable amount of the solvent MeOH was added as a negative control. After evaporation of MeOH with a gentle stream of nitrogen CYPRO remained as oily film on the bottom of the well plate. Finally, 5 mL FASW were added to each well of the controls.

For the H_2_O_2_ settlement experiments, a stock solution of 20 mM H_2_O_2_ in FASW was created from 30% stabilized H_2_O_2_ (Sigma-Aldrich, St-Louis, MO, United States). Four concentrations of H_2_O_2_ (1, 10, 100 and 500 µM) in 5 mL filtered artificial seawater were tested for their potential to induce settlement in *L. purpurea* larvae. For each of the three replicated controls and H_2_O_2_ treatments, three coral larvae were added to each well. Larval settlement behavior was examined under a dissecting microscope at the beginning of each experiment and after 12, 24, 36 and 48 h. All assays were performed under a 12 h alternating dark-light rhythm (starting in complete darkness) and kept at 27 °C over the entire length of the experiments using an incubator (Certomat BS-1, Sartorius AG, Göttingen, Germany). Larval behavior was differentiated into five categories: (i) swimming, (ii) attachment, (iii) `nonsense metamorphosis’, (iv) settlement (attachment and metamorphosis) and (v) mortality (disintegration). Larvae were designated “attached” if water movements created by a pipette motion (ca. 0.5 mL s^−1^, 1 mm diameter opening, 5 mm distance to the larvae) could not detach them. ‘Nonsense metamorphosis’ was indicated if an oral disk and mesenteries were clearly visible (typical flower shape), while settlement was determined if the flower-like recruits were also attached to the well or substrate.

### Confocal imaging

A Leica SP5 equipped with several lasers (argon, helium neon and 405 nm diode) including a multiphoton laser was used for obtaining confocal laser fluorescence microscopy images. To document CYPRO uptake, free-swimming coral larvae (negative control) and coral larvae exposed to CYPRO in the dark and collected at different settlement stages (12 h: attachment, 24–48 h: different stages of metamorphosis) were examined. Single and stacked shots (n = 50 slides) were taken using an excitation of 514 nm and an emission window of 550–570 nm for CYPRO detection (Figure S1). For the identification of GFPs, an excitation wavelength of 488 nm and an emission window of 500–520 nm was applied (Figure S1). Free-swimming coral larvae were supplemented with magnesium chloride (MgCl_2_), an additive that reduced movement and enabled clear examination under the microscope.

### Hydrogen peroxide production assays

The Amplex UltraRed hydrogen peroxide/peroxidase assay kit and the included Amplex UltraRed reagent (10-acetyl-3,7-dihydroxyphenoxazine) were used to measure the production of hydrogen peroxide (H_2_O_2_). A stock solution of 20 mM H_2_O_2_ was created from 30% stabilized H_2_O_2_ (Sigma-Aldrich, St-Louis, MO, United States) and used to develop a calibration curve (0, 0.01, 0.1, 0.25, 0.5, 1, 5, 10 and 20 µM H_2_O_2_; *cf.* Figure S4). For this purpose, 50 µL of an Amplex UltraRed working solution, consisting of 4.85 mL citric acid buffer (24.087 g sodium citrate dehydrate, 3.471 g citric acid, 1 L Milli-Q water, pH 6.0), 100 µL horseradish peroxidase (0.2 U mL^−1^) and 50 µL Amplex UltraRed (100 µM Amplex UltraRed), was mixed with 50 µL of each H_2_O_2_ dilution step. The calibration and the assays were performed in transparent 96-well plates (BRAND GmbH & Co. KG, Wertheim, Germany) using a Synergy H1 microplate reader (BioTek Instruments, Inc., Winooski, VT, United States) with the excitation wavelength set to 530 nm and the fluorescence emission wavelength recorded at 590 nm. In this context, fluorescent resorufin was produced in a 1:1 stoichiometry reaction between Amplex UltraRed and H_2_O_2_ in the presence of horseradish peroxidase. After calibration, different concentrations of CYPRO were tested for their potential to produce H_2_O_2_: (i) two concentrations of CYPRO (10 and 20 µg in 300 µL FASW) recorded for 36 h while applying a 12 h dark-light cycle (12 h each, starting with darkness); and (ii) four concentrations of CYPRO (1, 1.5, 5 and 10 µg in 300 µL FASW) recorded over a time period of 4 h under full white light. For this purpose, CYPRO was dissolved in UHPLC-grade MeOH and applied to the well bottoms at the respective concentrations. MeOH without CYPRO was used as blanks. After evaporation of the MeOH, 300 µL FASW was added to each well. All experiments and blanks were tested in triplicates. For H_2_O_2_ analysis, 50 µL Amplex UltraRed working solution were added to 50 µL of the respective sample/blank and the mixture was analyzed using the microplate reader with settings applied as mentioned above.

### Statistical analysis

Settlement behavior data were not normally distributed, thus all data points were tested together against the negative controls for significance (with the exception of those whose three replicates of one treatment were all 0) using a non-parametric Kruskal–Wallis test followed by a pairwise Dunn’s test with Bonferroni correction to perform multiple comparisons. Thus, the p-values indicate the difference between all three replicates of the different treatments (i.e., compound concentration) compared to the negative control, resulting in multiple p-values for the settlement behavior experiments. Statistical analysis was conducted with SPSS Statistics 26 (IBM, version 26).

## Supplementary Information

Supplementary Figures.

## Data Availability

All data are available in the main text or the supplementary materials.
