# Supplementary material for: Photodegradation of a bacterial pigment and resulting hydrogen peroxide release enable coral settlement
Source: Sci Rep. 2023 Mar 2;13:3562. doi: 10.1038/s41598-023-30470-w (PMC9981606; doi:10.1038/s41598-023-30470-w)
Supplement: Supplementary file 1 — Supplementary Figures. [file 41598_2023_30470_MOESM1_ESM.docx]

Supplementary Information for

**Photodegradation of a bacterial pigment and resulting hydrogen peroxide release enable coral settlement**

Lars-Erik Petersen, Matthias Y. Kellermann*, Laura J. Fiegel,

Samuel Nietzer, Ulf Bickmeyer, Doris Abele and Peter J. Schupp*

*Corresponding author: Matthias Y. Kellermann ([matthias.kellermann@uni-oldenburg.de](mailto:matthias.kellermann@uni-oldenburg.de)), Peter J. Schupp ([peter.schupp@uni-oldenburg.de](mailto:peter.schupp@uni-oldenburg.de))

**This PDF file includes:**

Figs. S1 to S4

| **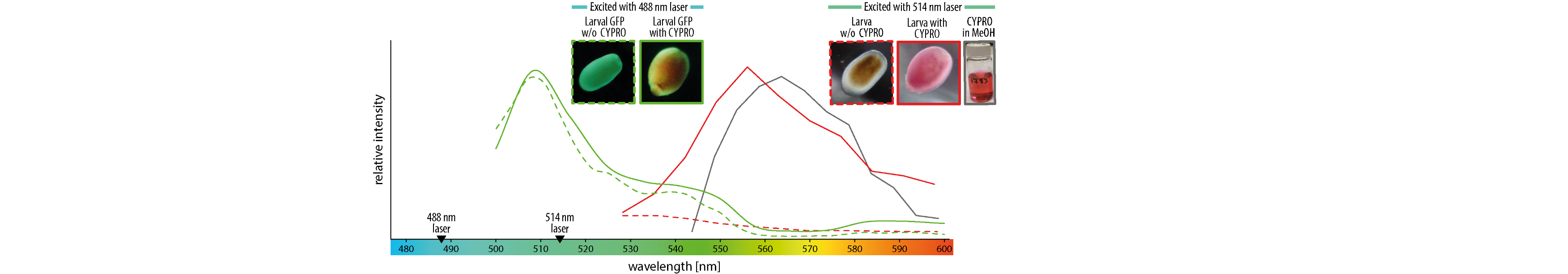** |
| --- |
| **Fig. S1. Emission spectra of *Leptastrea purpurea* larvae supplemented without (dashed line) and with (solid line) CYPRO**. Emission spectrum of purified CYPRO dissolved in MeOH is shown in gray. Excitation wavelength for GFP and CYPRO were determined using 488 nm and 514 nm, respectively. |

| 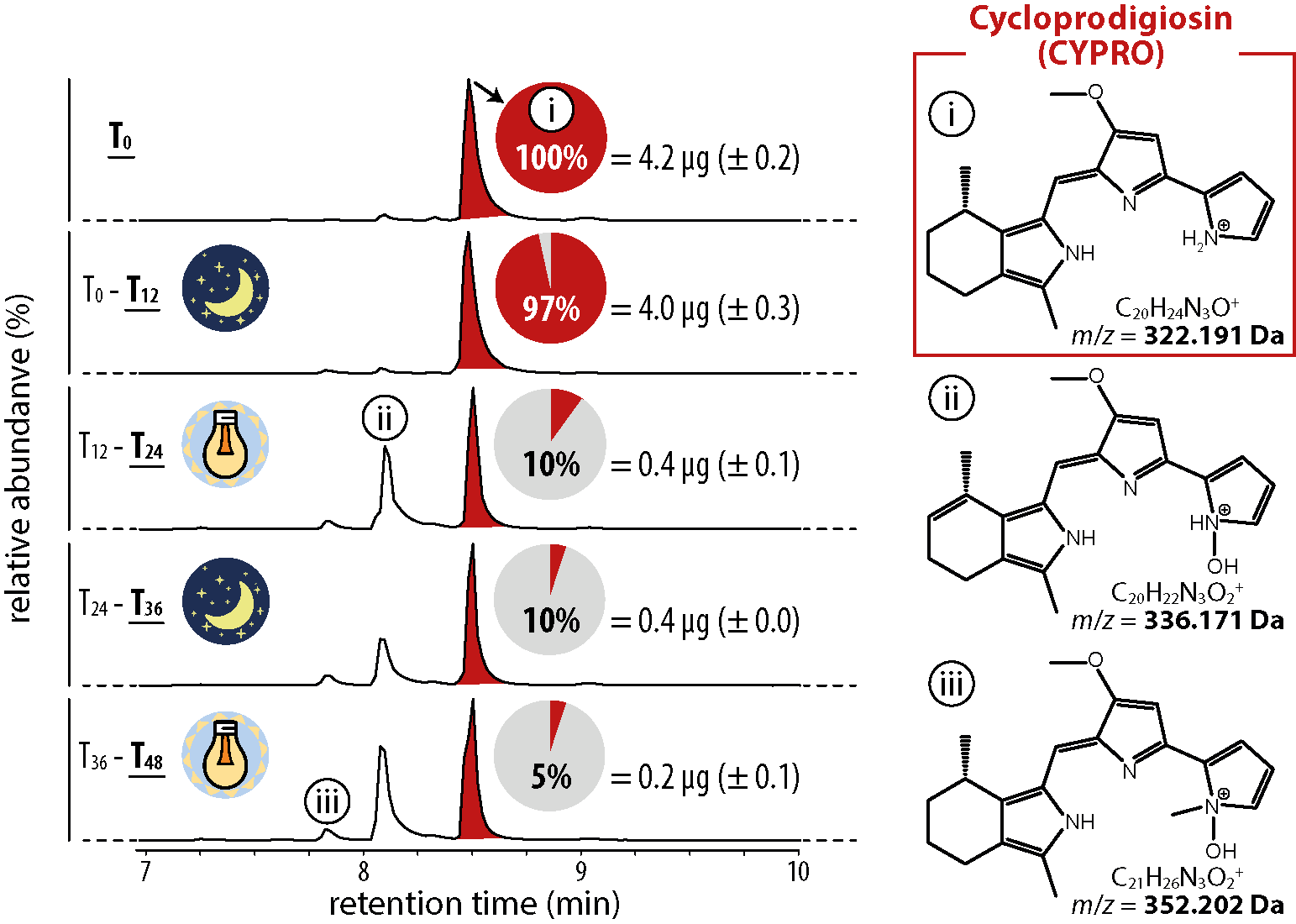 |
| --- |
| **Fig. S2. Sensitivity of CYPRO in response to oscillating light regimes.** While the concentration of CYPRO barely changed in the first 12 h of darkness, the following 12 h of light stress induced a drastic decrease of the red pigment down to 10%. Note that the exact molecular structure was only fully revealed for CYPRO *(28)*, the putative oxidized byproducts of CYPRO are based solely on its molecular mass and thus are still tentative. See also Petersen and colleagues *(28)* for more information about light sensitivity of CYPRO. |

| 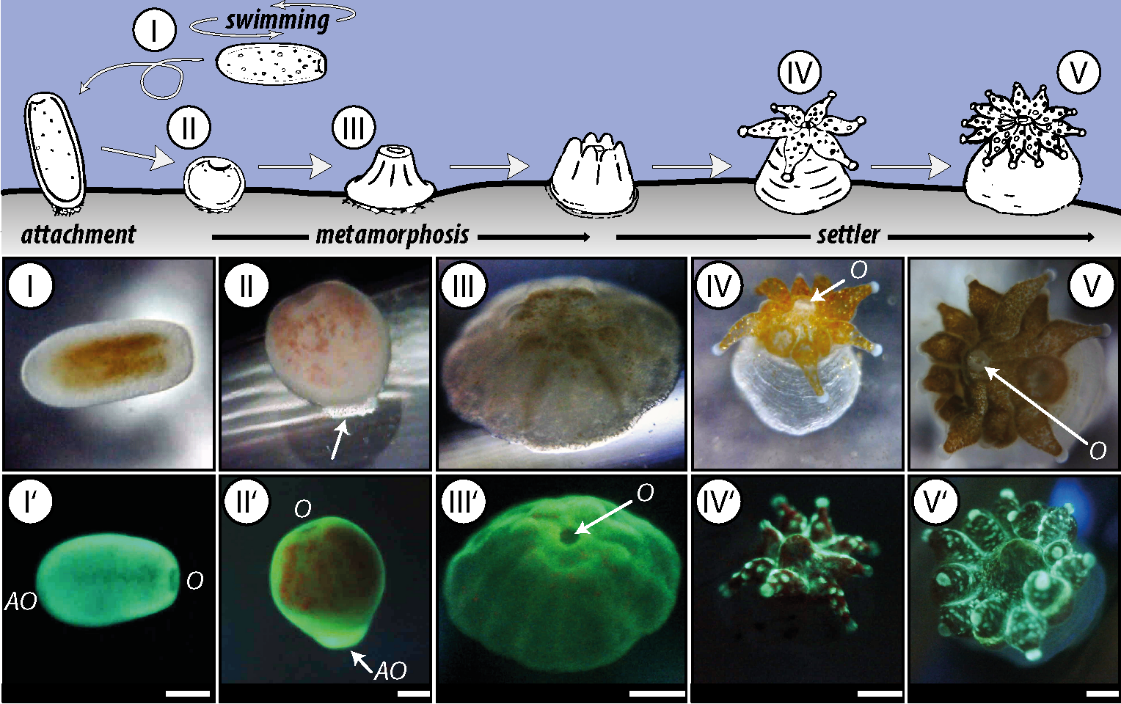 |
| --- |
| **Fig. S3. Early life stages of the scleractinian brooding coral *L. purpurea* in response to the bioactive morphogen CYPRO**. Sequential Roman numbers in the schematic drawing and the photographs below indicate different steps in larval/recruit development. Photographs were taken using white LED light, photographs with apostrophe were captured using blue light (wavelength: 430-460 nm) and a yellow barrier filter to visualize only the red and green fluorescence emitted either from the larvae/recruits and/or their associated symbionts. (**I, I’**) free-swimming planula larva; (**II, II’**) attached larva after 12 h experiment duration; (**III, III’**) attached and metamorphosed primary recruit after 48 h; (**IV, IV’**) self-sufficient polyp with six fully developed primary tentacles two weeks after settlement; (**V, V’**) polyp with in total 12 tentacles five weeks after settlement. Scale bar: 250 µm. Figure has been modified from Fiegel and colleagues *(40)*. |

| **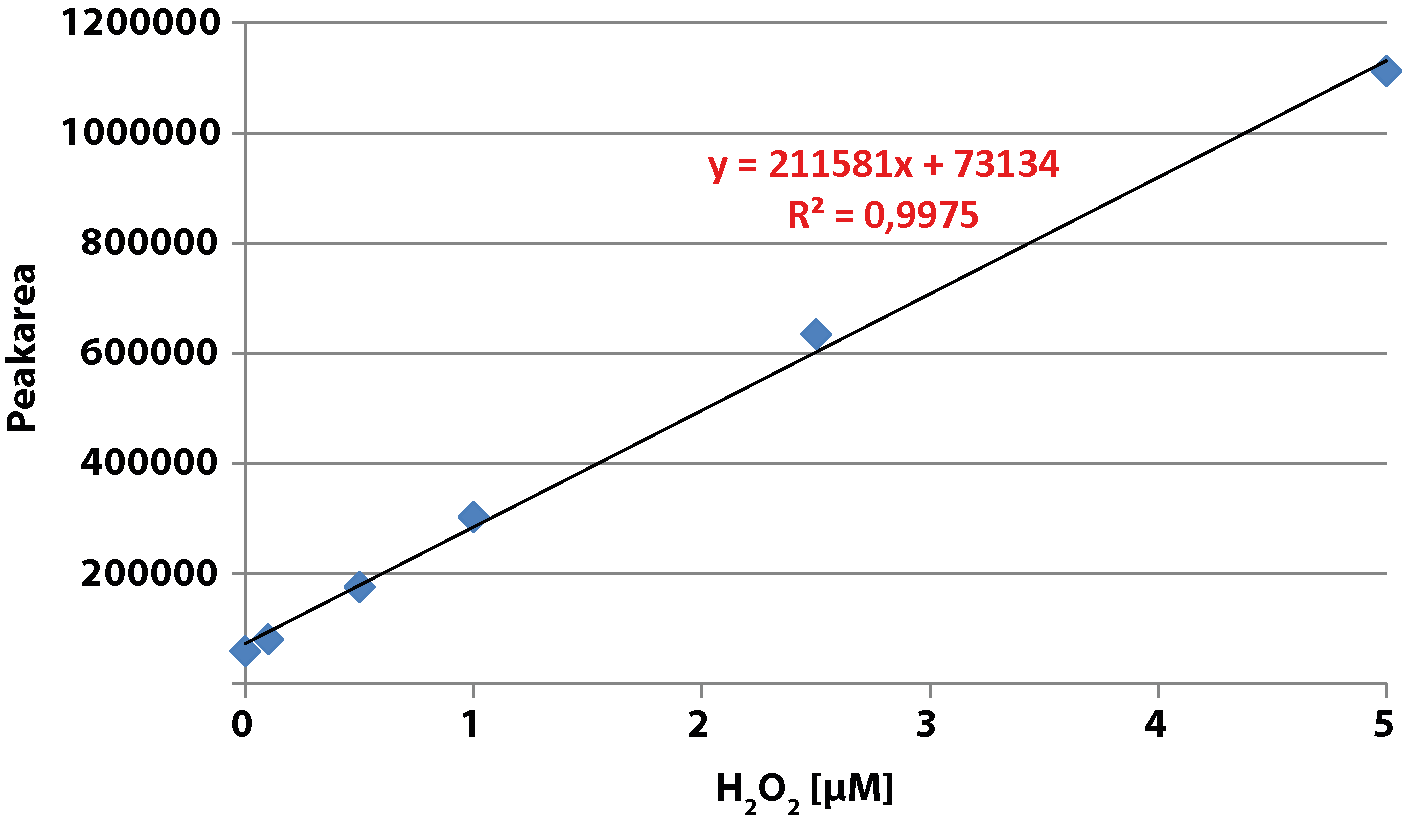** |
| --- |
| **Fig. S4. Calibration curve for H_2_O_2_ production assay.** 0, 0.01, 0.1, 0.25, 0.5, 1, 2,5 and 5 µM H_2_O_2_ were used. |
